# Characterization of the p53 Cistrome – DNA Binding Cooperativity Dissects p53's Tumor Suppressor Functions

**DOI:** 10.1371/journal.pgen.1003726

**Published:** 2013-08-15

**Authors:** Katharina Schlereth, Charlotte Heyl, Anna-Maria Krampitz, Marco Mernberger, Florian Finkernagel, Maren Scharfe, Michael Jarek, Ellen Leich, Andreas Rosenwald, Thorsten Stiewe

**Affiliations:** 1 Molecular Oncology, Philipps-University, Marburg, Germany; 2 Institute of Molecular Biology and Tumor Research, Philipps-University, Marburg, Germany; 3 Genome Analytics, Helmholtz Centre for Infection Research, Braunschweig, Germany; 4 Institute of Pathology, University of Würzburg, Würzburg, Germany; University of Washington, United States of America

## Abstract

p53 protects us from cancer by transcriptionally regulating tumor suppressive programs designed to either prevent the development or clonal expansion of malignant cells. How p53 selects target genes in the genome in a context- and tissue-specific manner remains largely obscure. There is growing evidence that the ability of p53 to bind DNA in a cooperative manner prominently influences target gene selection with activation of the apoptosis program being completely dependent on DNA binding cooperativity. Here, we used ChIP-seq to comprehensively profile the cistrome of p53 mutants with reduced or increased cooperativity. The analysis highlighted a particular relevance of cooperativity for extending the p53 cistrome to non-canonical binding sequences characterized by deletions, spacer insertions and base mismatches. Furthermore, it revealed a striking functional separation of the cistrome on the basis of cooperativity; with low cooperativity genes being significantly enriched for cell cycle and high cooperativity genes for apoptotic functions. Importantly, expression of high but not low cooperativity genes was correlated with superior survival in breast cancer patients. Interestingly, in contrast to most p53-activated genes, p53-repressed genes did not commonly contain p53 binding elements. Nevertheless, both the degree of gene activation and repression were cooperativity-dependent, suggesting that p53-mediated gene repression is largely indirect and mediated by cooperativity-dependently transactivated gene products such as CDKN1A, E2F7 and non-coding RNAs. Since both activation of apoptosis genes with non-canonical response elements and repression of pro-survival genes are crucial for p53's apoptotic activity, the cistrome analysis comprehensively explains why p53-induced apoptosis, but not cell cycle arrest, strongly depends on the intermolecular cooperation of p53 molecules as a possible safeguard mechanism protecting from accidental cell killing.

## Author Summary

The tumor suppressor gene p53 counteracts tumor growth by activating genes that prevent cell proliferation or induce cell death. How p53 selects genes in the genome to direct cell fate specifically into one or the other direction remains unclear. We show that the ability of p53 molecules to interact and thereby cooperate, influences which genes in the genome p53 is regulating. In the absence of cooperation, p53 only binds and regulates a limited ‘default’ set of genes that is proficient to stop cell proliferation but insufficient to induce cell death. Cooperation increases p53's DNA binding and enables context-dependent activation of apoptosis genes and repression of pro-survival genes which together triggers cell death. As the concerted effort of p53 molecules is needed, the threshold for cell killing is raised possibly to protect us from accidental cell loss. Thus, by shaping the genomic binding pattern, p53 cooperation fine-tunes the gene activity pattern to steer cell fate into the most appropriate, context-dependent direction. The genome-wide binding patterns of cooperating and non-cooperating p53 proteins generated in this study provide a comprehensive list of p53 binding sites as a resource for the scientific community to further explore mechanisms of tumor suppression by p53.

## Introduction

The prominence of the p53 gene in tumor suppression is emphasized by its unsurpassed mutation rate in cancer cells [1]. As a master regulatory transcription factor for anti-proliferative programs, p53 can decide cell fate in response to a broad range of stress stimuli, including DNA damage and oncogene activation [1], [2], . p53 prevents the accumulation of precancerous cells by activating genes involved in cell cycle arrest (e.g. *p21/CDKN1A*, *GADD45A*, *SFN*, *E2F7*) and apoptosis (e.g. *BAX*, *PMAIP1/NOXA*, *PUMA*) or repressing cell proliferation genes [5]. While gene activation is well-studied, the mechanism of p53-dependent target gene repression is still poorly understood and both direct and indirect models are discussed [5], [6]. On the one hand, p53 prevents genes from becoming activated by directly binding to promoters or distal enhancer elements - thereby competing with other activating transcription factors and components of the basic transcriptional machinery - or by recruiting histone-modifying enzymes with repressive functions such as mSin3A [5]. On the other hand, p53 indirectly represses proliferation genes by upregulation of several coding (*p21/CDKN1A*, *E2F7*) and non-coding RNAs (miR-34 family, lincRNA-p21) [7], [8], [9], [10], [11], [12].

Sequence specific DNA binding of p53 requires a DNA motif that consists of two decameric half-sites (RRRCWWGYYY; R = A/G, W = A/T, Y = C/T) separated by an optional spacer of additional base pairs to form a full-site [13]. Previous *in vitro* studies demonstrated that the central CWWG defines the torsional flexibility of the DNA and thus influences p53's binding affinity [14]. While a CATG sequence is flexible and therefore bound with high affinity, the other possible CWWG sequences are not [15]. In fact, it has been suggested that the inflexible CWWG sequences and spacer containing sites require a higher binding energy and therefore represent low affinity p53 binding sites [14], [15], [16], [17]. Interestingly, high affinity p53 motifs are specifically enriched among pro-arrest genes, whereas the promoters of pro-death targets predominantly contain low affinity sites [13], [16], [18]. Despite these biophysical differences between p53 binding sequences, it remains unclear at present how p53 molecularly distinguishes between distinct target genes to bind and activate a selected set.

**Figure 1:**
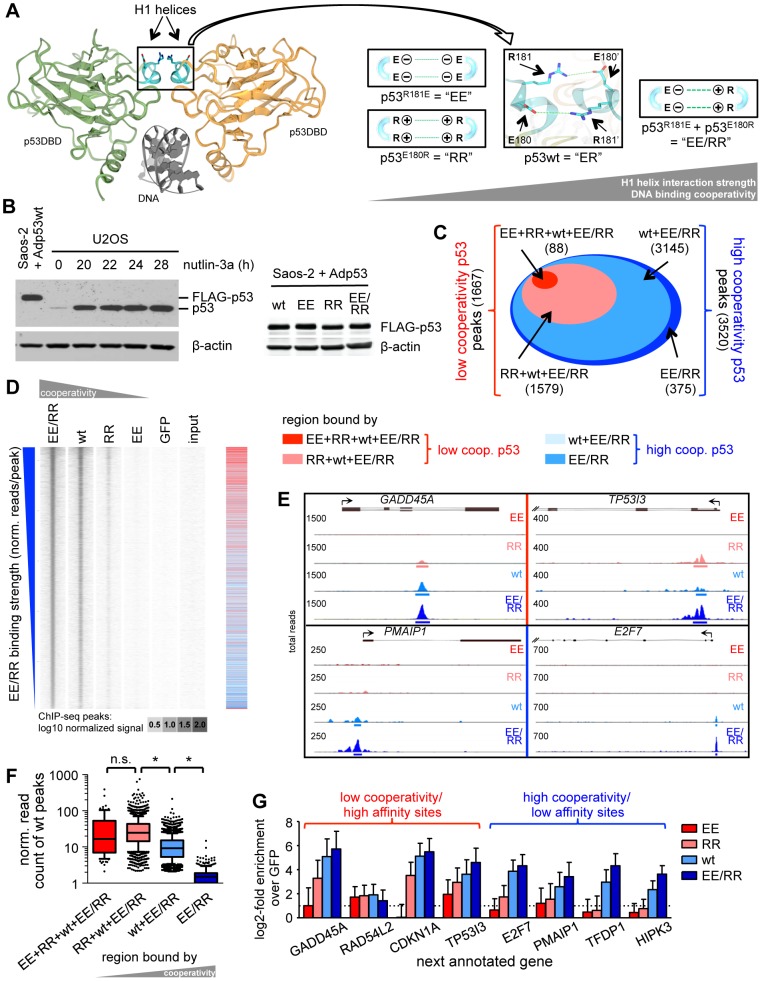
DNA binding cooperativity extends the p53 cistrome to low affinity binding sites. (*A*) *Left*: dimer of p53 DNA binding domains (green and yellow) on the DNA (gray) (Protein Data Bank ID code 2ADY) [20]. Highlighted in blue are the H1 helices. *Right*: Design of complementing p53 cooperativity mutants at glutamate E180 or arginine R181. (*B*) p53 Western Blot of U2OS cells treated for the indicated time with 10 µM nutlin-3a and Saos-2 cells infected for 18 hours with the indicated p53-expressing adenoviruses. β-actin is shown as a loading control. (*C*) Classification of p53 binding sites identified by ChIP-seq according to cooperativity. (*D*) Density blot of all p53 ChIP-seq peaks arranged in order of decreasing EE/RR binding strength. The heat map (right) depicts the classification illustrated in (*C*). (*E*) Strength of wild-type p53 binding to low and high cooperativity regions. Depicted is a box-and-whiskers blot with 10/90 percentiles and the median; outliers are plotted as dots. n.s.; not significant. *; p-value<0.001 (ANOVA-Tukeys honest significant differences based on log-transformed normalized read counts). (*F*) Genome browser views of p53 binding to selected low (top) and high (bottom) cooperativity regions. The numbers on the y-axis of each track represent the total number of overlapping reads. (*G*) Validation of ChIP-seq data by qPCR. Shown is the mean (±SD) log2-fold enrichment relative to the GFP control sample of two independent experiments with three qPCR replicates each.

Structurally, p53 proteins assemble into an asymmetric tetramer that can be described as a dimer of symmetric dimers. Tetramerization is mediated via the C-terminal oligomerization domains and further stabilized through interactions between neighboring DNA binding domains [19], [20]. In detail, oppositely charged amino acids (Glu180, Arg181) in the H1 helices of the DNA binding domains form an inter-molecular double salt bridge that enables adjacent p53 molecules to interact and cooperate when binding to DNA – a property known as DNA binding cooperativity (Fig. 1A) [21], [22], [23], [24]. Of note, cooperativity has been shown to be required for p53-induced apoptosis but not cell cycle arrest [24], [25]. Furthermore, somatic p53 mutations resulting in reduced cooperativity are found in cancer patients, germline cooperativity mutations segregate with cancer susceptibility in Li-Fraumeni syndrome families, and cooperativity mutant mice are highly cancer prone, indicating that DNA binding cooperativity is essential for proper tumor suppression [24], [25].

The aim of this study was to comprehensively characterize the impact of DNA binding cooperativity on all p53 binding sites in the genome (the p53 cistrome) by combined analysis of global DNA binding (ChIP-seq) and expression data. We demonstrate that high DNA binding cooperativity is crucial for the binding and transactivation of low affinity binding sites in pro-apoptotic genes with non-canonical and spacer-containing p53 motifs and also for p53-mediated repression of mitotic and pro-survival genes. Since both transactivation of genes with non-canonical response elements and p53-mediated gene repression are essential for p53-induced apoptosis, these data comprehensively explain why p53 molecules need to cooperate for cell killing as the basis for efficient tumor suppression.

## Results

### DNA binding cooperativity extends the p53 cistrome to low affinity binding sites

To explore the role of DNA binding cooperativity for the genome-wide binding pattern of p53, we comprehensively mapped the binding sites of p53 proteins in different cooperativity states by deep sequencing of immunoprecipitated chromatin (ChIP-seq). The p53 cooperativity mutation “EE” (p53^R181E^) causes four negatively charged glutamic acid residues to cluster at the H1 helix interaction interface which strongly destabilizes the intermolecular interactions and reduces DNA binding cooperativity [24]. Likewise, the mutation “RR” (p53^E180R^) brings four positively charged arginine residues together resulting in a similar destabilization and low degree of cooperativity. Importantly, combined expression of EE and RR (EE/RR) results in mixed tetramers in which one negatively and one positively charged H1 helix interact, resulting in a DNA binding cooperativity that slightly exceeds that of the wild-type (wt) (Fig. 1A). Combined expression of EE and RR therefore rescues the cooperativity defect of the EE and RR homotetramers. Following transfection in p53-negative Saos-2 cells all p53 variants were expressed at equal levels comparable to endogenous p53 in U2OS cells treated with the MDM2 inhibitor nutlin-3a (Fig. 1B) [26]. ChIP sequencing resulted for each sample in more than 30 M reads that were mapped to the genome. p53 binding peaks were called applying a stringent false discovery rate (FDR) of 10^−5^. Moreover, only peaks with a minimum number of 50 reads and 2-fold change versus GFP and input were considered as binding sites. These criteria ensured the identification of only reliably p53 bound regions. In detail, 88 peaks were determined as EE binding sites and additional 1579 sites were bound by RR, which together represent 1667 low cooperativity peaks (Fig. 1C). 3145 additional sites occupied by wild-type p53 together with 375 sites bound by EE/RR only, formed the 3520 high cooperativity peaks. Thus, the number of p53 binding sites rises with increasing DNA binding cooperativity as illustrated in the peak density plot (Fig. 1D). When binding sites were ranked according to decreasing EE/RR binding strength as a measure of binding affinity, EE and RR sites clustered at the top (see heatmap in Fig. 1D), indicating that only the high affinity p53 binding sites that were strongly bound by EE/RR or wild-type p53 were also bound by low cooperativity p53. Accordingly, the binding strength of wild-type p53 to high affinity (low cooperativity) sites was significantly stronger than to low affinity (high cooperativity) sites that were only occupied by wild-type p53 and/or EE/RR (Fig. 1E). These correlations between cooperativity and binding strength were also evident on the single gene level (Fig. 1F) and confirmed in independent validation experiments (Fig. 1G). In summary, the genome-wide ChIP analysis revealed that DNA binding cooperativity extends the number of p53 sites by enabling recruitment to low affinity sites.

### Cooperativity reduces the sequence specificity of p53 DNA binding

**Figure 2:**
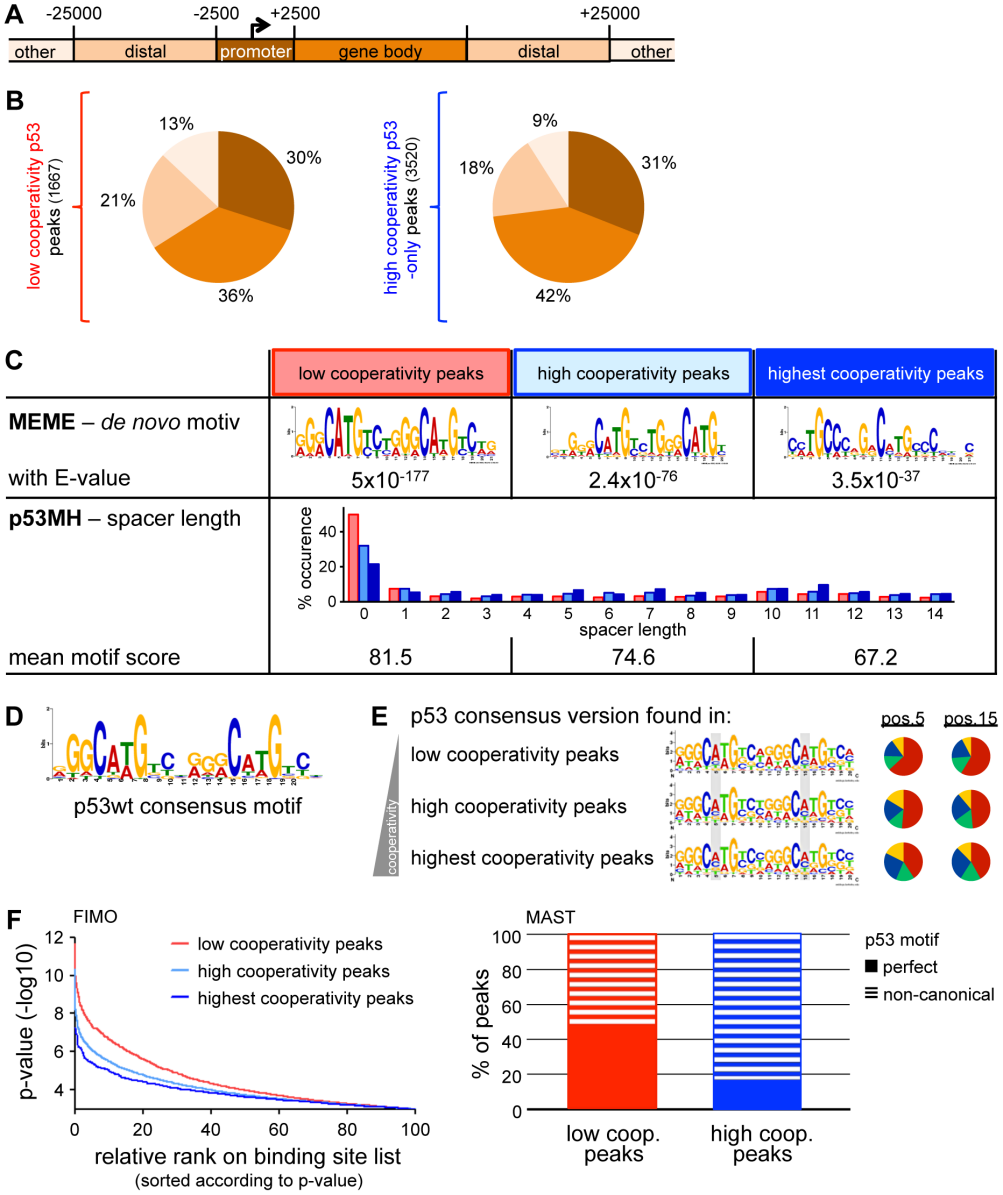
Cooperativity reduces the sequence specificity of p53 DNA binding. (*A*) Classification of genomic regions. (B) Distribution of p53 binding sites across the genome on the basis of the classification depicted in (*A*). (*C*) Motif analysis within low (first column), high (second column) and highest cooperativity p53 binding sites. The third column represents the subgroup of high cooperativity binding sites bound by EE/RR only. *Top*: *De novo* motif search by MEME-ChIP. Depicted is the top motif (lowest E-value). *Bottom*: Distribution of spacer lengths and mean motif score determined by the p53MH algorithm. (*D*) The p53 consensus motif generated from the top50 wild-type p53 binding sites in this study. (*E*) Low cooperativity mutants display a preference for CAWG at the CWWG core of the p53 consensus binding sequence. Motifs fitting to the p53 consensus sequence (*D*) within the three different cooperativity groups of binding sites defined in (*C*) were identified using FIMO and illustrated by WebLogo. The pie charts represent the base distribution at positions 5 and 15 of the consensus sequence (shaded in gray). (*F*) Low cooperativity sites confine better to the p53 consensus binding sequence than high cooperativity sites. *Left*: p53 binding sites in the indicated cooperativity groups were ranked according to similarity (p-value) to the p53 consensus binding sequence as determined with the FIMO algorithm. *Right*: Percentage of peaks containing perfect or non-canonical p53 motifs in low and high cooperativity regions, respectively, based on MAST analysis.

To explore the location of p53 binding sites in the genome, we divided the genome into the regions: gene body, promoter, distal and intergenic (Fig. 2A). p53 binding sites of both, low and high affinity regions were preferentially located directly at the promoter or within the gene body (above 70% in each group) and only rarely at further distance indicating that low and high cooperativity sites are distributed similarly across the genome (Fig. 2B).

As a previous ChIP-on-Chip analysis restricted to promoter regions of the genome suggested that the DNA binding cooperativity of p53 influences the sequence preference of p53 [24], we further characterized the p53 sequence motif in high and low cooperativity p53 binding sites of our genome-wide ChIP-seq dataset. A *de novo* motif search within all groups of p53 peaks - independent of the level of cooperativity - revealed a p53 motif with significant similarity to the consensus p53 motif (JASPAR database) (Fig. 2C). p53 motifs identified in the group of low cooperativity sites showed high uniformity (E-value 5×10^−177^) while motifs in high cooperativity sites were more divers (E-value 2.4×10^−76^) with the most variability in the subgroup of EE/RR-only bound peaks (E-value 3.5×10^−37^). In fact, the EE/RR-only motif was more similar to a p53 half-site than to a full-site. Furthermore, a search for p53 motifs on the basis of the wild-type p53 consensus from the present ChIP-seq analysis (Fig. 2D) revealed a strong preference for an A in the core CWWG sequences of each half-site that became less obvious with increasing cooperativity (Fig. 2E). To directly compare the quality of p53 motifs in the different cooperativity groups, we scored every single motif instance on the basis of similarity to the wild-type p53 consensus motif using two independent algorithms (Fig. 2F). Approximately 50% of the low cooperativity p53 binding sites matched perfectly to the consensus in contrast to less than 20% of the high cooperativity sites (Fig. 2F, right). In parallel, the mean motif score as determined by the p53MH algorithm [27] decreased with increasing cooperativity (Fig. 2C, bottom). Moreover, whereas spacer sequences were absent in about half of the motif instances in the low cooperativity peaks, 70 to 80% of the motifs identified in high cooperativity peaks contained spacers of variable length (Fig. 2C, bottom).

Together, the cistrome analysis suggests that p53 with low DNA binding cooperativity only binds to full-site p53 DNA motifs with high similarity to the consensus binding sequence. In contrast, motifs occupied by highly cooperative p53 only, show reduced similarity to the p53 consensus motif and comprise not only full but also half-sites separated by spacers of variable length. Thus, p53 requires high DNA binding cooperativity for binding to non-canonical p53 motifs.

### The requirement for DNA binding cooperativity separates the p53 cistrome according to function

**Figure 3:**
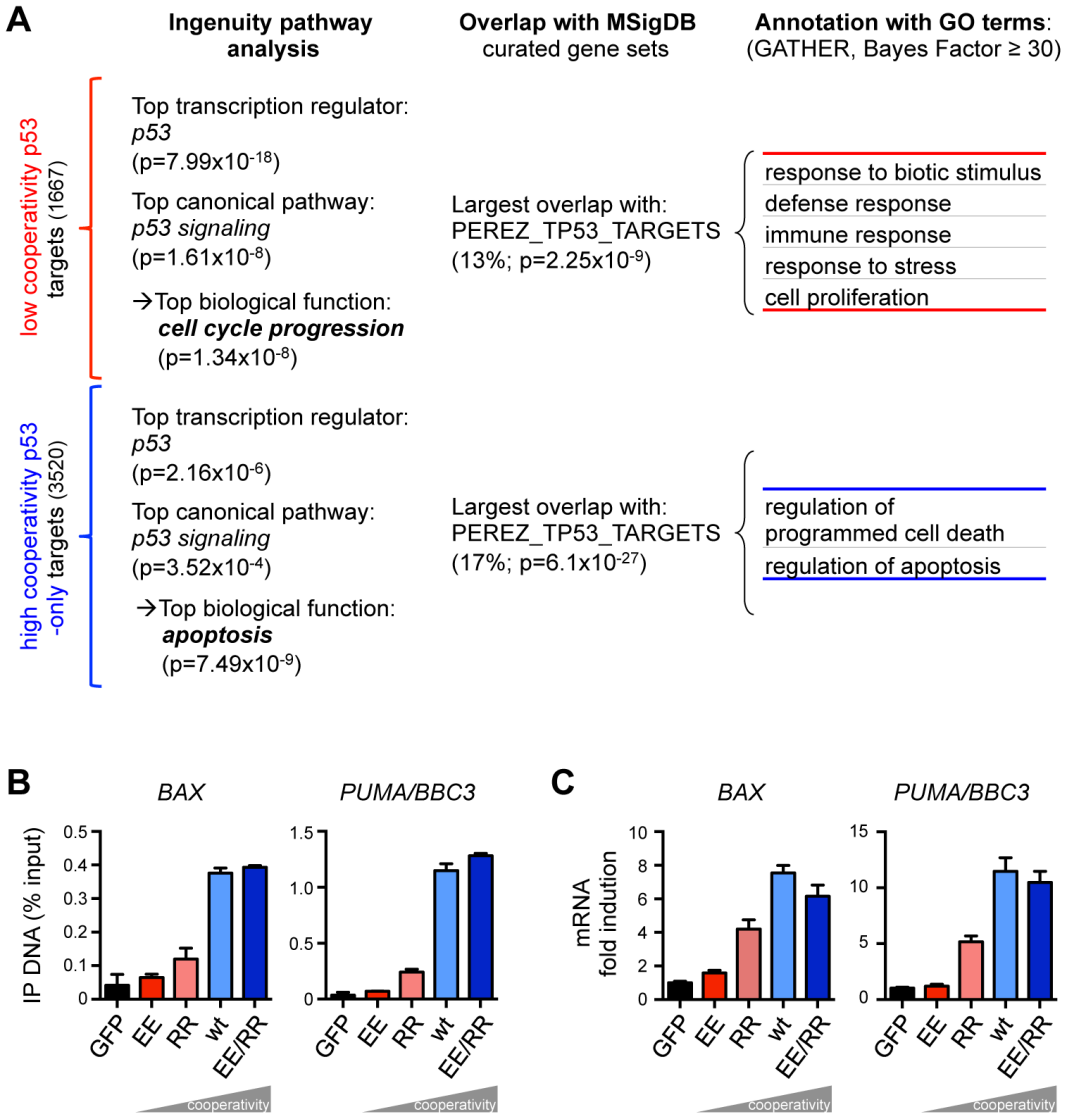
The requirement for DNA binding cooperativity functionally separates the p53 cistrome. (*A*) Functional annotation of the neighboring genes closest to the p53 binding sites by Ingenuity Pathway Analysis or MSigDB (the percentage denotes the proportion of genes that overlap). The overlap with the MSigDB gene set PEREZ_TP53_TARGETS was annotated with Gene Ontology (GO). Shown are the GO terms unique for either list. (*B*) Quantification of p53 binding to the *BAX* and *PUMA/BBC3* genes by ChIP-qPCR. Shown is the mean (±SD, n = 3) binding expressed in % of input chromatin. (*C*) RTqPCR quantification of *BAX* and *PUMA/BBC3* mRNA following expression of the indicated p53 variants. Shown is the mean (±SD, n = 3) mRNA expression normalized to GAPDH and the mock sample.

The genes closest to the p53 binding sites were functionally annotated using a combination of different algorithms. Expectedly, Ingenuity Pathway Analysis identified “p53” and “p53 signaling” as the most significant transcriptional regulator and canonical pathway, respectively, in both cooperativity groups (Fig. 3) which validates the two gene lists as significantly enriched for *bona fide* p53 target genes. Interestingly, the top biological function for low cooperativity target genes was cell cycle progression, in contrast to apoptosis for high cooperativity genes. The cooperativity-dependent difference in biological function was further confirmed by overlap analysis with gene sets in the Molecular Signature Database (MSigDB, Fig. 3) and functional annotation with Gene Ontology terms (Fig. 3). Both p53 binding site groups showed the strongest overlap with an experimentally defined set of p53-induced genes [28]. Importantly, genes with high affinity p53 sites were again annotated with cell proliferation, stress and immune responses and included well-characterized cell cycle arrest genes such as *CDKN1A* and *BTG2* [29], [30]. In contrast, genes with low affinity p53 peaks were associated with cell death, and apoptosis in particular, including critical pro-apoptotic genes. Cooperativity-dependent regulation of pro-apoptotic genes was validated for *BAX* and *PUMA/BBC3*, two well-established apoptotic target genes of p53 (Fig. 3B, C) [5]. Thus, the requirement for DNA binding cooperativity, which determines the affinity towards different p53 motifs, functionally separates the p53 cistrome into cell cycle regulation and apoptosis.

**Figure 4:**
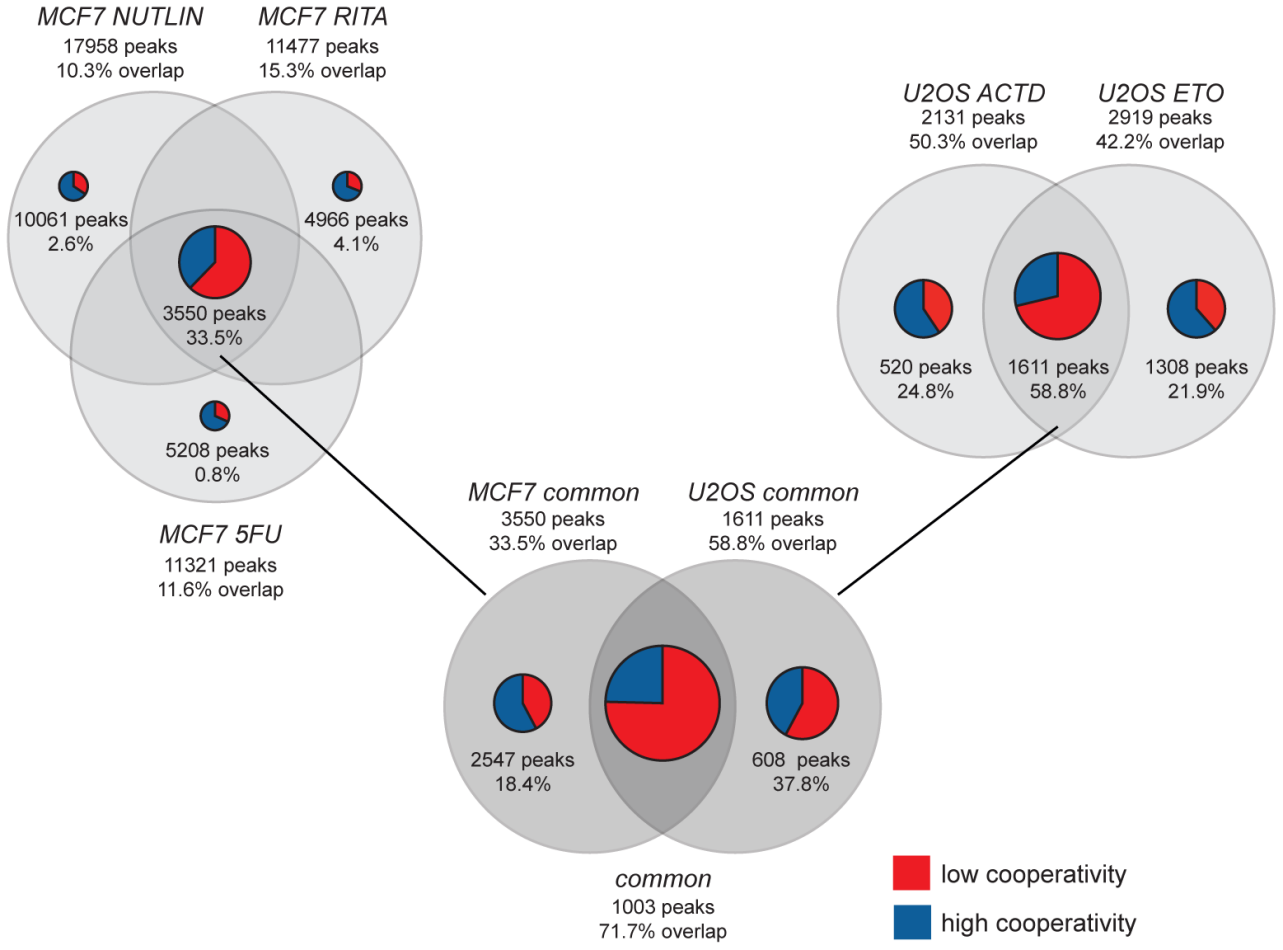
ChIP-seq meta-analysis identifies a role for cooperativity in the context-dependent fine-tuning of the p53 response. Venn diagrams illustrate the overlap of p53 binding peaks revealed in MCF7 and U2OS cells treated with the indicated p53-activating compounds (5-fluorouracil, 5FU; nutlin-3a; RITA; etoposide, ETO; actinomycin D, ACTD) [31], [32]. The number of binding peaks and the percentage of overlap with the total number of 5187 p53 binding sites identified in Saos-2 cells in our study are indicated. The total size of the pie charts reflects the degree of overlap with the p53 cistrome of Saos-2 cells, while blue and red sections illustrate the proportion of high and low cooperativity binding sites, respectively. For example, 50.3% of the 2131 p53 binding peaks identified in actinomycin D treated U2OS cells were also present in Saos-2 cells. 520 of these peaks were unique to actinomycin D treatment and 24.8% of them were present in Saos-2 cells. The majority (59.4%) of these were high cooperativity peaks. The treatment-independent sets of p53 binding peaks were denoted *MCF7 common* and *U2OS common*, respectively. The *common* peak set comprises the 1003 treatment-independent peaks that were identified in both MCF7 and U2OS cells.

The cistrome of wild-type p53 has been previously characterized in a number of different cell types under various p53-activating conditions [31], [32], [33]. To explore the impact of p53 DNA binding cooperativity in a broader context, we compared our data obtained from Saos-2 osteosarcoma cells to p53 ChIP-seq data obtained in the breast cancer cell line MCF7 treated with 5-fluorouracil (5FU) or MDM2 inhibitors (nutlin-3a, RITA) [32] and the osteosarcoma cell line U2OS treated with actinomycin D or etoposide [31] (Fig. 4). In both MCF7 and U2OS cells the p53 cistrome was strongly influenced by the type of p53-activating stimulus and only subsets of all binding peaks were bound in a treatment-independent manner: 3550 *MCF7 common* and 1611 *U2OS common* peaks,. Furthermore, the comparison of MCF7 and U2OS cells revealed a pronounced cell type-specificity of the p53 cistrome so that only 1003 *common* peaks were bound by p53 in both cells types. 719 (71.7%) of these *common* peaks were also present in Saos-2 cells, strongly supporting the hypothesis, previously raised by Nikulenkov et al. [32], that there is a ‘default set’ of p53 binding sites in the genome that is bound largely independent of treatment and cell type.

Many of the other p53 peaks that we identified in Saos-2 cells were also present in MCF7 or U2OS cells, but often only in one cell-type or following a specific treatment, as indicated in Fig. 4 by the percentage of overlap. When analyzing the relative proportion of low and high cooperativity peaks within the overlap, we found that the common ‘default set’ of binding peaks was mostly comprised of low cooperativity peaks, while the overlap with cell type- or treatment-specific peak sets showed a higher percentage of high cooperativity peaks. These data suggest that the ‘default program’ of p53 activation, that possibly functions as a first-line defense to genomic damage, does not require DNA binding cooperativity, while a fine-tuned p53 response, that integrates context-specific cues in a cell type- and stress-dependent manner, strongly relies on DNA binding cooperativity.

### Gene regulation by p53 is cooperativity-dependent

**Figure 5:**
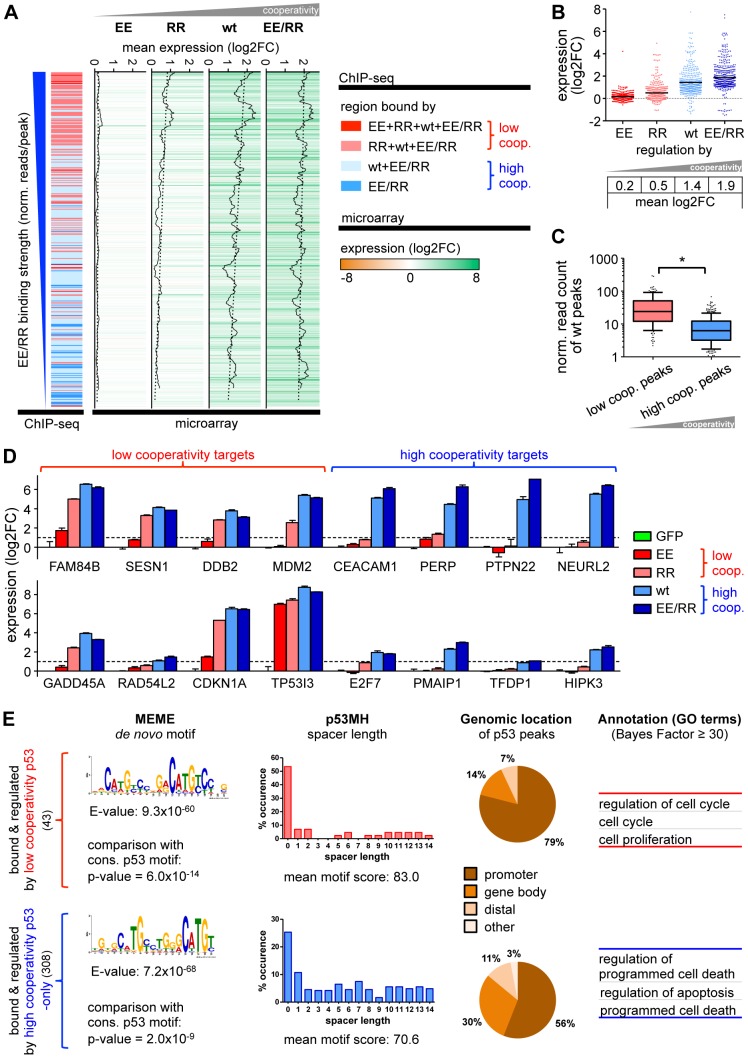
Gene regulation by p53 is cooperativity-dependent. (*A*) Correlation of the ChIP-seq data with the corresponding expression profiles revealed 351 differentially expressed genes with 489 distinct binding sites. Expression levels of these genes are depicted in a heat map ranked by decreasing EE/RR binding strength. The cooperativity classification of genes according to ChIP-seq is shown on the left as in Fig. 1D. Gene regulation by p53 increases with DNA binding cooperativity and correlates with DNA binding strength as shown in the walking average plot of expression for each p53 cooperativity mutant. (*B*) Expression of p53-bound and -regulated genes according to cooperativity. Shown is the log2-fold expression change. The black horizontal bar indicates the mean. (*C*) DNA binding strength (in reads/peak) of wild-type p53 to low or high cooperativity sites in differentially regulated genes. Depicted is a box-and-whiskers blot with 10/90 percentiles and the median; outliers are plotted as dots. *, p<0.001. (*D*) Validation of microarray results by RTqPCR analysis. Shown is the mean (±SD, n = 3) log2-fold expression change. (*E*) Motif search (MEME), spacer analysis (p53MH) and genomic classification of p53 binding sites in differentially regulated genes followed by functional annotation with GO terms as in Fig. 2 and 3.

To investigate the role of DNA binding cooperativity for gene regulation, the p53 cooperativity mutants were analyzed by microarray-based expression profiling in combination with ChIP-seq. 351 genes that were bound by at least one of the p53 versions were found to be differentially regulated by more than 2-fold (Fig. 5A, Supplemental Table S1). As shown above for the complete p53 cistrome (Fig. 1E), DNA binding cooperativity determined the binding strength also in the regulated part of the cistrome, i.e. the subset of p53-bound and -regulated genes (Fig. 5A,C). Interestingly, the vast majority of these genes (97%) was p53-induced and not repressed. Although the EE mutant was identified on a small number of genes by ChIP-seq, EE was no potent mediator of gene regulation. For all other p53 proteins the transactivation of individual genes directly correlated with binding strength and the average degree of regulation rose with increasing cooperativity (Fig. 5A,B). Gene regulation by the low cooperativity mutant RR was therefore confined to the subset of genes with high affinity binding sites whereas high cooperativity p53 showed additional regulation of low affinity targets (Fig. 5A). The correlation of DNA binding cooperativity with gene regulation was confirmed in validation experiments by RTqPCR for several genes (Fig. 5D), some of which were previously validated to be bound in a cooperativity-dependent manner (Fig. 1G). Interestingly, *RAD54L2*, which recruits all p53 cooperativity mutants to a similar extent, showed nevertheless cooperativity-dependent induction suggesting that the role of cooperativity extends beyond regulation of DNA binding and might affect transactivation by additional mechanisms. Furthermore, although *TP53I3* (*PIG3*) displays cooperativity-dependent recruitment to its binding site, it is a rare example of a target gene that was transactivated independently of cooperativity, indicating that low-level binding of p53 maximally activates some genes already. It is tempting to speculate that this exception is due to the peculiar binding of p53 to a polymorphic pentanucleotide microsatellite in the *TP53I3* promoter [34]. We conclude from these data that cooperativity not only increases the binding site spectrum of p53 but also gene regulation with respect to gene number and activation level.

We next explored potential sequence, positional and functional differences between low and high cooperativity binding sites in the regulated subset of the cistrome (Fig. 5E). *De novo* motif search discovered in both cooperativity groups a motif significantly resembling the p53 consensus binding motif. However, the motif was less perfect for the high cooperativity peaks and resembled more a half-site than a full-site. In line with this, more than 50% of the motifs in the low cooperativity group were spacer-free in contrast to only 26% in the high cooperativity group. Different from the genome-wide analysis, the genomic location of low and high cooperativity binding sites across the p53-regulated genes varied substantially. 79% of the low cooperativity sites were located within the promoter and only 14% within the gene body, in comparison to 56% and 30% of the high cooperativity sites, respectively. Importantly, functional annotation again revealed a separation of the bound and regulated genes into distinct gene ontology categories with low cooperativity target genes being annotated with cell cycle regulation and high cooperativity genes with cell death.

Together, the DNA binding cooperativity of p53 not only determines the number of genomic binding sites but also the number of regulated genes, the vast majority of which are p53-induced instead of repressed. Moreover, not only DNA binding strength but also the level of transactivation correlates directly with the degree of cooperativity indicating that cooperativity enhances p53's impact on the cistrome and transcriptome. Most importantly, p53-regulated genes with low and high cooperativity binding sites differ significantly in their biological function. Transcriptional activation of the apoptosis program requires a higher degree of intermolecular cooperation likely as a safeguard against accidental elimination of cells.

### Clinical relevance of cooperativity for the survival of breast cancer patients

**Figure 6:**
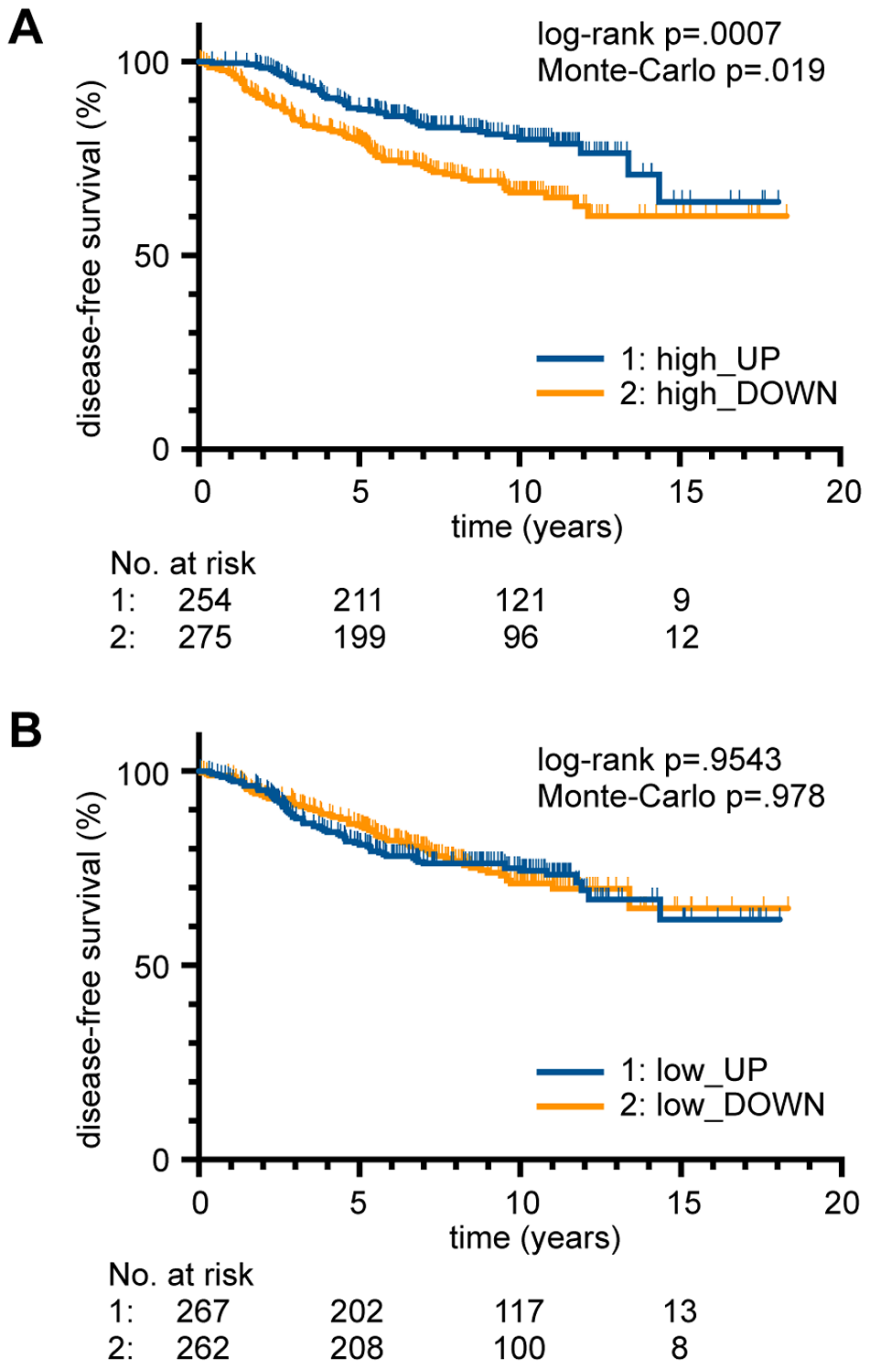
Role of DNA binding cooperativity for the survival of breast cancer patients. (*A,B*) Kaplan-Meier survival estimates for breast cancer patients [35], [37] stratified on the basis of (*A*) high and (*B*) low cooperativity target gene expression (Suppl. Table S4). Patients with an upregulated expression (high_UP; low_UP) were plotted against patients with a downregulation (high_DOWN; low_DOWN). The number of patients at risk in each group at a given time point is indicated below the plots. p-values from log-rank test and Monte-Carlo simulations are indicated in the figure.

It was previously shown by gene expression profiling that p53 mutant and wild-type breast cancer samples are molecularly distinct and that p53-dependent transcriptional signatures not only predict p53 status but also disease-specific survival [35]. The correlation of superior survival with upregulated expression of p53-induced genes was validated in multiple datasets from independent patient cohorts [36]. Considering the role of DNA binding cooperativity for the regulation of functionally distinct classes of p53 target genes, we explored whether expression of low and high cooperativity genes affects patient survival to a similar extent. Using published microarray-based expression data from breast cancer patients [35], [37] we employed a previously described gene set enrichment analysis (GSEA) approach to assess whether the gene expression profile of a patient is enriched in low and/or high cooperativity p53 target genes [38]. Kaplan-Meier curves showed that upregulated expression of high cooperativity target genes was significantly associated with superior survival (Fig. 6A). Surprisingly, no such correlation was observed for low cooperativity genes (Fig. 6B). These data suggest that not all p53 target genes are equally potent in tumor suppression and that only high cooperativity genes are able to prolong the survival of breast cancer patients.

### p53-dependent gene repression requires high DNA binding cooperativity

**Figure 7:**
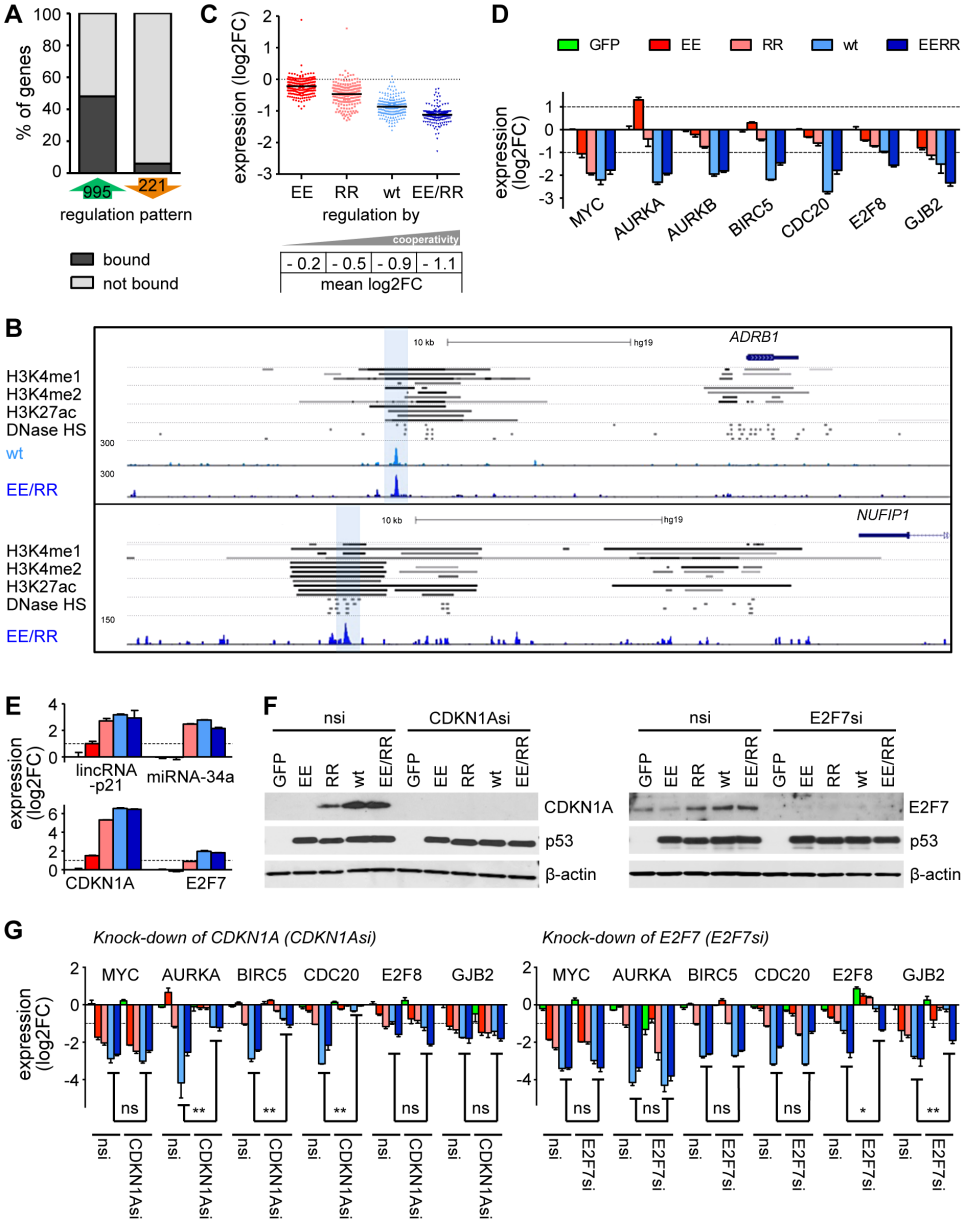
p53-dependent gene repression requires high DNA binding cooperativity. (*A*) The percentage of differentially regulated genes with or without a p53 binding site in our ChIP-seq dataset. (*B*) Genome browser views of distal p53 binding peaks that overlap with enhancer sites marked by H3K4 mono- and dimethylation, H3K27 acetylation and DNase I hypersensitivity (HS) in four different cell lines (GM12878, HUVEC, NHEK, HSMM; named in the order of appearance). The bars represent regions of statistically significant signal enrichment [UCSC browser tracks; 71]. (*C*) Expression of p53-downregulated genes according to cooperativity. The black horizontal bar indicates the mean. (*D*) Validation of the microarray results by RTqPCR analysis. (*E*) Expression analysis of indicated genes by RTqPCR analysis. (*E–G*) *CDKN1A* and *E2F7* are transactivated in a cooperativity-dependent manner (RTqPCR, *E*; immunoblot, *F*) and mediate p53-dependent repression of target genes (RTqPCR, *G*). nsi, non-silencing control siRNA. All bar graphs in this figure show the mean (±SD, n = 3) log2-fold expression change. Two-tailed Mann-Whitney *U* test: *, p<0.05; **, p<0.01. Bar colors are as indicated in (*D*).

In contrast to half of the p53-induced genes (476/995), less than 10% of the repressed genes (13/221) contained a p53 binding peak in the vicinity (Fig. 7A, Supplemental Table S2). In two repressed genes a p53 binding peak mapped to a distal enhancer element as defined by H3K4 mono- and dimethylation, H3K27 acetylation and DNase I hypersensitiviy (Fig. 7B) suggesting that p53 can mediate repression through interfering with distal enhancer activity as previously shown for mouse embryonic stem cells [39], [40], [41]. However, most of the downregulated genes did not contain a p53 binding site. Surprisingly, the level of downregulation nevertheless correlated with DNA binding cooperativity (Fig. 7C). While only three genes (*KLF6*, *MYC*, *UTP15*) were identified to be repressed by the low cooperativity mutant RR, wild-type p53 and EE/RR robustly repressed (mean 2-fold downregulation) multiple genes associated with mitotic progression (e.g. *AURKA*, *AURKB*, *CDC20*, *CCNB1*/*2*), survival (*BIRC5*) and developmental regulation (TGFβ and WNT signaling) (Fig. 7D, Supp. Table S2). We conclude that p53-mediated gene repression displays an even higher dependence on cooperativity than gene activation.

The lack of p53 binding peaks in the vicinity of most repressed genes suggested that p53-dependent repression is largely indirect. As the level of downregulation was shown to be dependent on the level of cooperativity, we predicted that downregulation is mediated by p53 target genes which are induced in a cooperativity-dependent manner. Several possible candidates that have previously been implicated in p53-mediated repression such as lincRNA-p21, miR-34a, *CDKN1A* and *E2F7* were all induced by p53 [7], [8], [9], [10], [12], but only *E2F7* showed a cooperativity-dependent expression pattern on the mRNA level (Fig. 7E). Although *CDKN1A* was transactivated in a cooperativity-independent manner, its gene product p21 showed a clear cooperativity-dependent induction on the protein level (Fig. 7E,F). To interrogate the role of these proteins for p53-mediated gene repression, we examined the effect of *CDKN1A* or *E2F7* depletion (Fig. 7F). The knock-down of *CDKN1A* did not prevent repression of *MYC*, *E2F8* or *GJB2*, but had a slight de-repressive effect on *AURKA* and *BIRC5* and resulted in complete de-repression of *CDC20* (Fig. 7G). Upon depletion of *E2F7* some genes were unaffected (*MYC*, *AURKA*, *BIRC5*, *CDC20*) whereas repression of *E2F8* and *GJB2* was strongly reduced (Fig. 7G). Additional bioinformatic analysis with Ingenuity Pathway Analysis and GeneXplain [42] in terms of p53-downregulated genes revealed a significant enrichment of transcription factor binding sites (Sp1, SMAD3, NF-Y) and common upstream regulators (YY1, FOXM1) as well as additional miRNAs (miR-34, miR-145, miR-200) several of which have been previously implicated in transcriptional repression by p53 [43].

Since p53 engagement of a repressive effector network comprising cell cycle inhibitors, transcriptional repressors, miRNAs and long non-coding RNAs is largely cooperativity-dependent, DNA binding cooperativity is therefore - despite the striking underrepresentation of p53-repressed genes in the p53 cistrome - nevertheless a major determinant of both gene activation and repression.

## Discussion

Cooperative DNA binding by p53 is known to be essential for p53-mediated cell death and cooperativity mutations in cancer patients suggest a role for tumor suppression [24], [44]. This is further supported by a selective apoptosis defect and cancer susceptibility of cooperativity mutant mice [25]. Here we used p53 H1 helix mutants in genome-wide DNA binding and expression analyses to comprehensively profile the role of DNA binding cooperativity for p53's function. In line with previous data showing that DNA binding of p53^R181E^ (EE) is hardly detectable [22], [24], we identified only a very small number of 88 EE binding sites in the genome compared to 4812 binding sites for wild-type p53 (Fig. 1C). Although the cooperativity-reducing p53^E180R^ (RR) mutation resulted in a similar DNA binding defect as the EE mutation when studied *in vitro* using recombinant proteins purified from *E. coli* [22], the DNA binding defect of RR expressed in mammalian cells was weaker and resulted in a cistrome of 1667 binding sites comprising mostly perfect consensus-like full-site motifs in genes enriched for cell cycle regulators. Importantly, while both EE and RR homotetramers have a more or less reduced cooperativity, they complement each other efficiently to yield EE/RR heterotetramers with a cooperativity higher than wild-type p53. This increases the efficiency of cooperative DNA binding and enables binding to a larger spectrum of 5188 sites enriched for non-canonical, spacer-containing p53 motifs in, for example, pro-apoptotic genes (Fig. 2 and 3). Together, these data prove that DNA binding cooperativity is a crucial modulator of p53's genome-wide binding pattern (cistrome) with important functional relevance for cell fate determination.

Previous ChIP-seq studies of activated wild-type p53 have identified approximately 1800 to 2900 significant binding sites [31], [32], which is comparable to the number of 1667 peaks bound by low cooperativity p53. Direct comparison of ChIP-seq data from different cells treated with multiple p53-activating drugs further revealed that the default set of p53 binding sites common to most cell types and independent of the type of activating stimulus largely comprises low cooperativity sites (Fig. 4). In contrast, binding sites that were bound by p53 in a cell-type and stress-specific manner were enriched in high cooperativity sites (Fig. 4), suggesting that fine-tuning the p53 response in a context-specific manner relies on DNA binding cooperativity.

On the DNA side, the interaction of p53 with a certain DNA motif is largely influenced by the central CWWG sequence even though the WW dinucleotide is not directly contacted by p53 [15], [19], [20]. As proper binding of the p53 tetramer to DNA requires bending of the DNA, different affinities of CWWG sequences can be explained by differences in bending flexibility [14], [20], [45]. As CATG is the most flexible CWWG sequence, intermolecular cooperation of p53 monomers is likely dispensable, while efficient binding to the more rigid non-CATG may require higher bending forces that depend on energetic stabilization provided by strong H1 helix interactions [24], [44]. Consistent with high affinity binding of p53 to CATG, we identified a specific enrichment of central CAWG sequences among the high affinity sites that were bound irrespective of cooperativity (Fig. 2E). In contrast, in line with lower affinity binding of p53 to CAAG, CTTG or CTAG, these non-CATG sequences showed a stronger dependence on cooperativity (Fig. 2E). Together, H1 helix interactions allow p53 molecules to cooperate to provide sufficient energy required for bending and binding a larger variety of sequences in the genome.

Importantly, there is growing evidence that such non-canonical, low affinity binding sites contribute substantially to p53's function [18]. First of all, considerable transactivation was observed at non-canonical half-sites (single decamers) and three-quarter-sites, some of which were originally classified as biologically relevant response elements (REs) in, for example, the pro-apoptotic target genes *PIDD* and *APAF1* [17]. Moreover, REs in many other functionally important pro-apoptotic genes show on average less similarity to the p53 consensus sequence and a lower degree of evolutionary conservation associated with higher sequence diversity than most prototypic cell cycle target genes such as *CDKN1A* [13], [16], [46]. Another example is the *VEGFR1* gene promoter, which contains a single nucleotide polymorphism that generates a non-canonical, functional p53 half-site thereby integrating the VEGF system into the p53 transcriptional network [47]. In fact, it is discussed that weak p53 REs have a selective advantage compared with high-affinity p53 binding sites as they could allow better fine-tuning of responses through the regulation of p53 protein levels, specific post-translational modifications or cofactors that modulate DNA binding affinity [18], [44], [48]. Crosstalk between p53 and the estrogen receptor in regulating *VEGFR1* provides a prominent example for the functional dependence on cooperation of p53 with other transcription factors for maximal activation of such non-canonical response elements [49]. Cooperativity therefore dramatically expands the p53 transcriptional network allowing the engagement of target genes that - likely as a safeguard - require a higher degree of stress or damage for activation.

The integrated analysis of ChIP-seq with expression profiling data revealed that less than 10% of the p53-bound genes were regulated by p53 (Fig. 5). This is in line with other studies [31], [33], [50], [51], and indicates that p53 binding to DNA is often not sufficient to induce transcription. Secondary stimuli or co-factors are needed, possibly in a stress or cell type specific manner, to induce a permissive chromatin state as previously suggested for single p53 target gene promoters [52]. While binding sites of low and high cooperativity p53 showed a similar distribution across the genome and were in 70–80% located in the promoter region or gene body (Fig. 2A,B), functional binding events that resulted in expression changes were distributed differently. While binding sites regulated by low cooperativity p53 were mainly enriched in the promoter region of genes, binding sites regulated by high cooperativity p53 were also frequently observed in the gene body (Fig. 5E). As binding of p53 to the regulatory promoter is more likely to have a direct effect on transcription than binding to a site further downstream of the transcriptional start site, this finding is consistent with the hypothesis that low affinity, non-canonical binding sites primarily function in cooperation with other transcription factors to fine-tune gene expression in response to context- or tissue-specific stimuli [18].

Interestingly, our analysis on the role of DNA binding cooperativity for patient survival showed a remarkable difference between low and high cooperativity genes. While upregulated expression of high cooperativity target genes correlated with a good clinical outcome, expression of low cooperativity target genes was surprisingly not correlated with distinct patient survival (Fig. 6). This indicates that low and high cooperativity genes are clinically not equivalent. Although low cooperativity genes comprise the default program of target genes activated in most cell types in a stimulus-independent manner, only the activation of high cooperativity genes is able to prolong patient survival. Together these data strongly emphasize the clinical relevance of DNA binding cooperativity for the anti-cancer activity of p53.

Intriguingly, a number of studies from both breast cancer patients and mice have found a wild-type p53 status to be associated with an inferior clinical response to chemotherapy compared to tumors with mutant p53 [53],[54],[55],[56]. For example, in *MMTV-Wnt1* driven mouse mammary tumors p53 wild-type tumor cells can evade an apoptotic chemotherapy response by undergoing arrest, followed by secretion of senescence-associated cytokines that can stimulate proliferation and relapse [53]. Given the functional separation of the p53 cistrome into high cooperativity genes with proapoptotic function and low cooperativity genes involved in cell cycle arrest, it is tempting to speculate that the expression ratio of high versus low cooperativity genes might determine the clinical response to chemotherapy in p53 wild-type tumors. While activation of high cooperativity genes is expected to prolong the long-term survival of the patient by supporting the apoptotic chemotherapy response, activation of low cooperativity genes leading to senescence might even be counter-productive and promote relapse. In fact, there was a trend - although not statistically significant - that in patients without upregulation of high cooperativity genes expression of low cooperativity genes was associated with an inferior survival (data not shown). Although it still remains to be investigated whether cooperativity has a similar impact on patient survival in other tumor entities, it is intriguing that the DNA binding cooperativity of p53 is not only crucial for preventing tumor development [25] but also appears to have a clinical impact on the survival of cancer patients under therapy.

Overall 221 genes, significantly enriched for mitotic and developmental regulators, were robustly repressed in response to p53 activation (Fig. 7). This is consistent with numerous studies that have established repression of cell cycle regulatory genes as a function of p53 [5]. For example, p53 robustly downregulates the *MYC* oncogene for induction of cell cycle arrest [57]. Surprisingly, in contrast to half of all activated genes, only 13 of 221 repressed genes showed p53 binding in the ChIP-seq experiment (Fig. 7A). Although some studies have reported direct binding of p53 to non-canonical p53 response elements in repressed genes, convincing genome-wide data supporting direct p53 binding as a general mechanism of repression is missing [5], [6]. Of note, interference of p53 with distal enhancer elements has been described to mediate repression of stem cell specific genes in murine embryonic stem cells [39]. We can confirm p53 binding to distal enhancers in two cases (*ADRB1*, *NUFIP1*), but overall the mechanism does not seem to play a prominent role in our cell model suggesting cell type specificity (Fig. 7B).

Interestingly, despite the absence of p53 binding events at repressed genes, the degree of repression was nevertheless dependent on cooperativity (Fig. 7). Importantly, p53-mediated repression of anti-apoptotic genes and oncogenes is crucial for p53-induced apoptosis [12], [58]. As cooperativity-reducing mutations result in apoptosis deficiency [24] and impair both the transactivation of important pro-apoptotic genes and the repression of a large set of genes, it is conceivable that cooperativity-dependent repression contributes to the pro-apoptotic function of p53. As p53 is not directly binding to most of the downregulated genes (Fig. 7A), an indirect mechanism involving p53-mediated transactivation of genes with repressor functions might be underlying repression mechanistically. In support of this, mice carrying a mutation in the p53 transactivation domain were reported as strongly impaired not only for transactivation but also repression [59]. Furthermore, a number of p53-activated genes including *CDKN1A* and *E2F7* have been implicated in repressing a variety of p53-regulated genes [7], [8], [9]. Consistently, we identified many E2F target genes as repressed by p53 and confirmed the requirement of *E2F7* and *CDKN1A* for the repression of mutually exclusive sets of genes (Fig. 7F,G). As both, E2F7 and p21, are induced in a cooperativity-dependent manner (Fig. 7F), their regulation could contribute to the cooperativity-dependent repression of at least a subset of p53-downregulated genes.

Furthermore, p53 has been implicated as a master regulator of miRNAs expression and processing [43]. The by far best studied group of p53-activated miRNAs is the miR-34 family that targets many mitotic genes contributing to senescence and apoptosis induction [10], [60], [61]. Other miRNAs induced by p53 such as miR-145 or the miR-200 family have been implicated as inhibitors of *MYC* and important developmental genes, respectively [43]. Our bioinformatics analysis of the cooperativity-dependently repressed genes revealed several miRNAs as potential upstream regulators, amongst others miR-34, miR-145 and miR-200. In addition, p53 was shown to repress genes indirectly by upregulating the large intergenic non-coding RNA lincRNA-p21, which is believed to interact with chromatin modifying complexes to silence target genes [12]. We observe induction of lincRNA-p21 in our study but do not see a major impact of cooperativity on lincRNA-p21 expression, excluding this lincRNA as a cause of cooperativity-dependent gene repression.

In principle, cooperativity-dependent gene repression in the absence of direct p53 binding to the repressed target promoters could alternatively indicate that cooperativity mutations affect other aspects of p53 function apart from DNA binding. While cooperativity mutations - different from many hot-spot mutations - do not affect the overall folding of the DNA binding domain [22], it is known that amino acids E180 and R181 are engaged in p53 interactions with ASPP family proteins that stimulate p53-transactivation of pro-apoptotic target genes [62], [63]. Furthermore, it has been described that association of p53 with promoter-specific cofactors like Sp1, SMAD3, NF-Y and YY1 results in gene repression [5], [6]. We applied both a sequence-based promoter analysis and a search for common upstream regulators of the p53-repressed genes and identified a significant enrichment of all these factors. Whether the interaction of Sp1, SMAD3, NF-Y and YY1 with p53 is dependent on cooperativity has so far not been explored. A future analysis of the interactome of p53 cooperativity mutants might therefore reveal additional insight into the effect of cooperativity mutations on gene repression.

In summary, our combined genome-wide analysis of DNA binding and gene expression using a set of p53 mutants with reduced and increased cooperativity reveals DNA binding cooperativity as a major modulator of the p53 cistrome. In particular the use of high cooperativity p53 enabled the compilation of a comprehensive set of p53 binding sites including many non-canonical response elements that have previously not been profiled. Interestingly, cooperativity is revealed to be not only important for p53 binding to non-perfect response elements but also for p53-mediated gene repression. Since both transactivation of non-canonical response element and p53-mediated repression are crucial for p53's pro-apoptotic activity, this strengthens the concept that the requirement for intermolecular p53 cooperation provides a novel safeguard mechanism protecting against the accidental activation of apoptosis as the most final, irreversible cell fate decision possible.

## Materials and Methods

### Cell culture, RNA interference and viral transduction

Saos-2 and U2OS cells were cultured in Dulbecco's modified Eagle's medium (Life Technologies) supplemented with 10% fetal bovine serum (PAA) and penicillin/streptomycin (Life Technologies) using standard conditions and procedures. siRNAs were purchased from Dharmacon and transfected with Lipofectamine RNAiMAX (Life Technologies) according to the manufacturer's protocol. Generation and use of recombinant adenoviruses for wild-type p53 and p53^R181E^ and p53^E180R^ have been described [24].

### Chromatin immunoprecipitation (ChIP)

Saos-2 cells were infected with adenovirus encoding GFP (as a control) or GFP together with wild-type or mutant p53. Cells were fixed 18 hours after infection in fresh 1% paraformaldehyde (PFA) for 10 min at room temperature (RT). Unreacted PFA was quenched by adding glycine to a final concentration of 125 mM for 5 min at RT. Cells were washed twice with ice-cold PBS and scraped from the dishes in ice-cold phosphate buffered saline supplemented with proteinase inhibitor (Complete, Roche). Cells were pelleted (700×g for 5 min at 4°C) and lysed at a concentration of 2×10^7^ cells/ml in SDS Lysis Buffer (1% SDS, 10 mM EDTA, 50 mM Tris pH 8.1) supplemented with proteinase inhibitor. Cells were sonicated on ice in 1 ml aliquots 5 times at 30% power for 10 sec followed by a 50 sec pause with a SONOPLUS sonifier with sonotrode MS72 (Bandelin electronics, Germany). Agarose gel electrophoresis confirmed shearing of crosslinked DNA into a smear in the range of 200–800 bp. After centrifugation at 10,000×g for 10 min at RT supernatants were diluted 1∶10 with Dilution Buffer (0.01% SDS, 1.1% Triton X-100, 1.2 mM EDTA, 16.7 mM Tris-HCl pH 8.1, 167 mM NaCl) and after 1 h of pre-clearing 1% input was removed from each sample and proteins were precipitated with p53 DO-1 antibody over night at 4°C. Mock-chromatin was immunoprecipitated from cells infected with GFP only. Complexes were bound to Protein G magnetic beads (Fast Flow, GE healthcare) for 2 h at 4°C and washed once with Low Salt Immune Complex Wash Buffer (0.1% SDS, 1% Triton X-100, 2 mM EDTA, 20 mM Tris-HCl pH 8.1, 150 mM NaCl), once with High Salt Immune Complex Wash Buffer (0.1% SDS, 1% Triton X-100, 2 mM EDTA, 20 mM Tris-HCl pH 8.1, 500 mM NaCl), once with LiCl Immune Complex Wash Buffer (0.25 M LiCl, 1% IGEPAL-CA630, 1% deoxycholic acid (sodium salt), 1 mM EDTA, 10 mM Tris-HCl pH 8.1), and twice with TE (10 mM Tris-HCl, 1 mM EDTA, pH 8.0) for about 5 min at 4°C. Complexes were eluted with Elution buffer (1% SDS, 0.1 M NaHCO_3_) for 20 min at 800 rpm at RT. Crosslinks were reversed at 65°C in 200 mM NaCl overnight followed by RNase A (37°C, 30 min) and Proteinase K digestion (45°C, 2 h). DNA was purified using the PCR Purification Kit (Qiagen) and DNA concentration was measured with PicoGreen (PicoGreen dsDNA Quantitation reagent, Molecular Probes). The enrichment was verified by qPCR for known binding sites. For primer sequences see Supplemental Table S3.

### ChIP-seq

For each sample, a single library was sequenced once on an Illumina GA IIx (ChIP-Seq Sample Preparation Kit, Cluster Generation Kit v2, 36-Cycle Sequencing Kit v3) and twice on an Illumina HiSeq2000 (TruSeq SR Cluster Kit v3 - cBot - HS, TruSeq SBS Kit v3 - HS - 50 cycles) system.

#### Data analysis

In order to keep all mapping comparable, only the first 36 bp of each read were used for further analysis. Reads were aligned to the *Homo sapiens* genome retrieved from Ensembl revision 65 with Bowtie 0.12.7 [64], using the parameter setting: ‘-k 1 -n 2 -e 70 -m 1’ to retrieve only uniquely matching reads with a low number of mismatches allowed. As a compromise between filtering for PCR induced artifacts and the saturation of the library complexity due to our read depth, read start positions with more than 28 reads were limited to exactly 28 reads. The resulting numbers of unique, perfectly matching reads were 34.3 M for the input, 37.6 M for the GFP control, 88.6 M for wild-type p53, 31.2 M for EE, 84.1 M for RR and 100.4 M for EE/RR.

Peak calling was performed separately on each sample using only the GA IIx sequencing run, using PARTEK Genomics Suite 6.6 (St. Louis, MO, USA). The significance threshold for the identification of enriched regions in the p53 samples was set to an FDR of <0.00001 and a *P*-value vs. GFP and input control of *P*≤0.05. An interval union of peaks from all samples was build and peaks were annotated with the next gene as judged by distance to the gene's most 5′ transcription start site. All gene annotations were retrieved from Ensembl revision 65. To enable comparison between the samples, combined tag counts from all sequencing runs were formed, and normalized to 1 million mapped reads. p53-presence was considered independently of the peak calling by ranking the conditions at each peak by their normalized read count and looking for at least a two fold difference between the ranks. Depending on the first rank showing such a difference to the previous, we designated the top one, two, three genotypes as binding. If no such ‘gap’ was found, we considered all genotypes as present. Binding profiles of p53 at selected genomic regions were visualized using PARTEK Genomics Suite 6.6 (St. Louis, MO, USA).

#### Motif discovery

Peaks were analyzed for enriched sequence motifs with MEME-ChIP [65]. Briefly, MEME-ChIP randomly selects 600 input sequences, which are then trimmed (central 100 bp) before entering the MEME algorithm. The discovered motifs were compared with known motifs from the JASPAR database using TOMTOM [66]. In addition, all identified peak sequences were analyzed with the spacer-tolerant p53MH algorithm for spacer length and the top scoring p53 full-site [27]. Further motif search and quality assessment was performed with the FIMO [67] and MAST [68] algorithm. More cis-regulatory factors were identified by Ingenuity Pathway Analysis (Ingenuity Systems) and GeneXplain [42].

#### Functional annotation

Pathway and biological process analyses were performed with Ingenuity Pathway Analysis (Ingenuity Systems) and the gene sets of the Molecular Signatures Database (MSigDB) v3.0 [69] and GATHER [70].

#### Comparison with ChIP-seq data from the literature

Previously published p53 ChIP-seq data were retrieved from the NCBI Gene Expression Omnibus (GSM545807, GSM 545808 and GSM545809, [31]) and supplemental data [32]. Peak data were extracted and lifted over to human genome assembly 19 (hg19). As reference genome we used the human genome assembly GRCh37 for all analyses (http://www.ncbi.nlm.nih.gov/projects/genome/assembly/grc/human/index.shtml). Peak lists were subsequently intersected to obtain the number of common peaks in two or more ChIP-seq analyses. Peaks were considered common if they overlap by at least one bp.

#### Gene Set Enrichment Analysis, Kaplan-Meier estimates and Monte Carlo simulations

To assess whether high or low cooperativity target genes are relevant for patient survival, a bioinformatics strategy based on Gene Set Enrichment Analysis [38], [69] was used to analyze gene expression datasets from two separate breast cancer studies [35], [37]. In detail, each gene expression dataset was mean-centered across samples in log scale and genes were ranked according to their expression level relative to the dataset mean as described [38]. To define high and low cooperativity gene sets the p53-bound and -regulated genes from Supplemental Table S1 were grouped into two sets based on the cooperativity classification of their p53 binding peak (Supplemental Table S4). Genes that had both low and high cooperativity peaks were considered as low cooperativity genes. Subsequently, for both the high and low cooperativity gene set enrichment scores were calculated for each tumor patient expression profile as described [38], [69]. The obtained enrichment scores were used to calculate Kaplan-Meier survival estimates for each gene set by separating patients into two groups, based on whether the obtained enrichment score was positive or negative. Statistical significance was assessed using the log-rank test. Furthermore, we performed Monte-Carlo simulations with 10,000 randomly generated gene signatures of equal size and used the obtained Kaplan-Meier estimates to calculate p-values.

### RNA isolation, RTqPCR and expression profiling

RNA isolation and cDNA synthesis were performed using the RNeasy Mini Kit (Qiagen) and SuperScript VILO cDNA Synthesis Kit (Life Technologies). miRNA isolation was performed using the mirVana miRNA Isolation Kit (Life Technologies) followed by reverse transcription using the TaqMan miRNA Reverse Transcription Kit (Life Technologies). Gene expression was quantified by RTqPCR using SYBR Green Jumpstart Taq ReadyMix (Sigma) or TaqMan MicroRNA Assays (U6 snRNA, miR-34a; Life Technologies) on a LightCycler 480 (Roche). Expression data were normalized to *GAPDH* or *U6 snRNA* and the mock sample using the ΔΔCt method. For primer sequences see Supplemental Table S3.

#### cDNA microarrays

cRNA of the Saos-2 cells expressing different p53 versions was prepared and hybridized to an oligonucleotide microarray (Human Genome U133 Plus 2.0 Array, Affymetrix) according to the manufacturers' instructions. The obtained expression signals were processed with the GeneChip Expression Console Software (Affymetrix) using the MAS 5.0 Data Processing Protocol. The expression fold change of each p53 version vs. GFP control cells was calculated and log2-transformed.

### SDS-PAGE, immunoblotting

Cells were lysed in 50 mM Tris-HCl pH 8.0, 150 mM NaCl, 5 mM EDTA pH 8.0, 2% Nonidet P-40 and the total protein concentration was quantified by Bradford assay. Samples were separated by SDS–PAGE and transferred onto nitrocellulose membranes (GE healthcare). After blocking with 10% non-fat dry milk, membranes were probed with antibodies specific for p53 DO-1 (gift from B. Vojtesek), CDKN1A (C-19, Santa Cruz), E2F7 (H-300, Santa Cruz), or β-actin (AC15, Abcam). Enhanced chemiluminescence (Thermo Scientific) or fluorescence (Odyssey Infrared Imaging System, LI-COR) was used for detection.

### Data access

ChIP-seq data have been deposited in the EBI ArrayExpress archive (http://www.ebi.ac.uk/arrayexpress/) and are accessible through the accession numbers E-MTAB-1394 (Username: Reviewer_E-MTAB-1394; Password: 0vmiovxP)

Microarray data sets have been deposited in the EBI ArrayExpress archive (http://www.ebi.ac.uk/arrayexpress/) and are accessible through the accession number E-MTAB-1403 (Username: Reviewer_E-MTAB-1403; Password: srkaaska)

## Supporting Information

Table S1 List of 489 p53-bound genes showing a p53-dependent expression change by more than 2-fold for at least one of the p53 variants. Shown are the location of the p53 binding peak, the distance to the next neighboring gene, normalized read count per peak, classification of peak localization according to gene structure, classification according to cooperativity group and log2-fold expression change versus GFP-control.
(XLSX)

Table S2 List of 221 genes showing a p53-dependent downregulation by more than 2-fold for at least one of the p53 variants. Shown are the log2-fold expression change versus GFP-control and the presence or absence of a p53 binding peak in the genomic neighborhood.
(XLSX)

Table S3 Primer sequences for RTqPCR and ChIP-qPCR.
(XLSX)

Table S4 List of p53-bound genes showing a p53-dependent expression change by more than 2-fold versus GFP-control for at least one of the p53 variants. Genes are divided into low and high cooperativity genes based on ChIP-seq results (Table S1). Genes with a low cooperativity peak were denoted as low cooperativity target genes, genes with a high cooperativity peak as high cooperativity target genes. Genes with both low and high cooperativity binding peaks were classified as low cooperativity target genes.
(XLSX)
